# *rac*-Dimethyl 2-(1*H*-pyrrole-2-carboxamido)­butane­dioate

**DOI:** 10.1107/S1600536811007148

**Published:** 2011-03-02

**Authors:** Le Zheng, Fang Hu, Xiang Chao Zeng, Kai Ping Li

**Affiliations:** a Department of Chemistry, Jinan University, Guangzhou, Guangdong 510632, People’s Republic of China

## Abstract

The title compound, C_11_H_14_N_2_O_5_, was synthesized by condensation of (*RS*)-2-amino­succinic acid dimethyl ester with 2-trichloro­acetyl­pyrrole at room temperature. The amide group is twisted by 7.4 (1)° from the plane of the pyrrole ring. In the crystal, mol­ecules are linked by inter­molecular N—H⋯O hydrogen bonds into chains extending along the *c* axis.

## Related literature

For the bioactivity of pyrrole derivatives, see: Fabio *et al.* (2007 ▶); Banwell *et al.* (2006 ▶). For related structures, see: Zeng *et al.* (2010 ▶); Li *et al.* (2009 ▶); Liu *et al.* (2006 ▶).
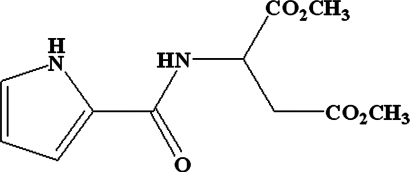


## Experimental

### 

#### Crystal data

C_11_H_14_N_2_O_5_
*M* *_r_* = 254.24
Monoclinic, 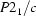

*a* = 9.1387 (8) Å
*b* = 15.2715 (11) Å
*c* = 9.6238 (9) Å
β = 105.750 (9)°
*V* = 1292.69 (19) Å^3^
*Z* = 4
Mo *K*α radiation
μ = 0.10 mm^−1^
*T* = 293 K
0.48 × 0.26 × 0.21 mm

#### Data collection

Oxford Gemini S Ultra area-detector diffractometer
Absorption correction: multi-scan (*CrysAlis PRO*; Oxford Diffraction, 2010 ▶) *T*_min_ = 0.952, *T*_max_ = 0.978
5286 measured reflections
2534 independent reflections
1563 reflections with *I* > 2σ(*I*)
*R*_int_ = 0.032

#### Refinement

*R*[*F* ^2^ > 2σ(*F* ^2^)] = 0.059
*wR*(*F* ^2^) = 0.163
*S* = 1.05
2534 reflections
165 parameters
H-atom parameters constrained
Δρ_max_ = 0.18 e Å^−3^
Δρ_min_ = −0.21 e Å^−3^

### 

Data collection: *CrysAlis PRO* (Oxford Diffraction, 2010 ▶); cell refinement: *CrysAlis PRO*; data reduction: *CrysAlis PRO*; program(s) used to solve structure: *SHELXS97* (Sheldrick, 2008 ▶); program(s) used to refine structure: *SHELXL97* (Sheldrick, 2008 ▶); molecular graphics: *SHELXTL* (Sheldrick, 2008 ▶); software used to prepare material for publication: *SHELXTL*.

## Supplementary Material

Crystal structure: contains datablocks I, global. DOI: 10.1107/S1600536811007148/cv5056sup1.cif

Structure factors: contains datablocks I. DOI: 10.1107/S1600536811007148/cv5056Isup2.hkl

Additional supplementary materials:  crystallographic information; 3D view; checkCIF report

## Figures and Tables

**Table 1:** Hydrogen-bond geometry (Å, °)

*D*—H⋯*A*	*D*—H	H⋯*A*	*D*⋯*A*	*D*—H⋯*A*
N1—H1*A*⋯O1^i^	0.86	1.96	2.804 (3)	167
N2—H2*A*⋯O1^i^	0.86	1.99	2.845 (3)	176

Symmetry code: (i) 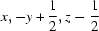.

**Fig. 1.**
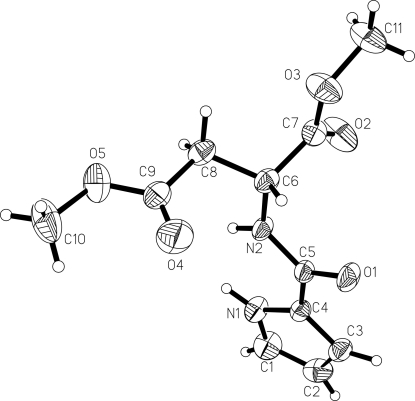
The molecular structure of (I) with the atom-numbering scheme. Displacement ellipsoids are drawn at the 30% probability level.

**Fig. 2.**
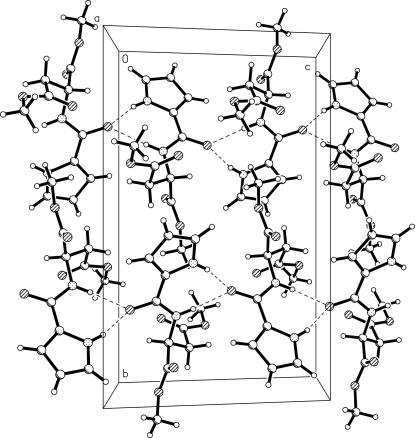
A portion of the crystal packing viewed approximately along the a axis. Dashed lines indicate hydrogen bonds.

## supplementary crystallographic information

### Comment

Pyrrole derivatives show various biological activities, for instance, antitumor activity (Banwell *et al.*, 2006). Some of them are known as metabotropic receptor antagonists (Fabio *et al.*, 2007). Herewith we present the title compound (I), which is a new pyrrole derivative.

In (I) (Fig. 1), all bond lengths and angles are normal and correspond to those observed in 1-benzyl-*N*-methyl-1*H*-pyrrole-2-carboxamide (Zeng *et al.*, 2010) and 3-(1-ethyl-1*H*-pyrrole-2-carboxamido) propionic acid monohydrate (Li *et al.*, 2009). In the crystal structure, enantiomorphous molecules are linked by intermolecular N—H···O hydrogen bonds (Table 1) into chains extended along the *c* axis (Fig. 2).

### Experimental

The hydrochloric acid salt of (*RS*)-2-aminosuccinic acid dimethyl ester (0.99 g, 5 mmol) and 2-trichloroacetylpyrrole (1.27 g, 6 mmol) were added to acetonitrile (12 ml), followed by the dropwise addition of triethylamine (1.4 ml). The mixture was stirred at room temperature for 12 h. After the reaction mixture was filtered, the filtrate was evaporated *in vacuo*, and then the residue was chromatographed over silica gel using EtOAc-petroleum ether (3:7 *v*/*v*) as eluting solvent and the title compound (I) was obtained as a light yellow solid (72.3% yield). Monoclinic crystals suitable for X-ray analysis (m.p. 384 K) grew over a period of five days when the EtOH solution of I was exposed to the air at room temperature.

### Refinement

All H atoms were positioned geometrically [C—H 0.93-0.98Å,  N—H 0.86 Å] and refined using a riding model, with *U*_iso_ = 1.2-1.5 *U*_eq_ of the parent atom.

### Crystal data

**Table:**

C_11_H_14_N_2_O_5_	*D*_x_ = 1.306 Mg m^−^^3^
*M**_r_* = 254.24	Melting point: 384 K
Monoclinic, *P*2_1_/*c*	Mo *K*α radiation, λ = 0.71073 Å
*a* = 9.1387 (8) Å	Cell parameters from 1891 reflections
*b* = 15.2715 (11) Å	θ = 3.5–29.4°
*c* = 9.6238 (9) Å	µ = 0.10 mm^−^^1^
β = 105.750 (9)°	*T* = 293 K
*V* = 1292.69 (19) Å^3^	Prism, light yellow
*Z* = 4	0.48 × 0.26 × 0.21 mm
*F*(000) = 536	

### Data collection

**Table:**

Oxford Gemini S Ultra area-detector diffractometer	2534 independent reflections
Radiation source: fine-focus sealed tube	1563 reflections with *I* > 2σ(*I*)
graphite	*R*_int_ = 0.032
φ and ω scans	θ_max_ = 26.0°, θ_min_ = 3.5°
Absorption correction: multi-scan (*CrysAlis PRO*; Oxford Diffraction, 2010)	*h* = −11→7
*T*_min_ = 0.952, *T*_max_ = 0.978	*k* = −15→18
5286 measured reflections	*l* = −11→11

### Refinement

**Table:**

Refinement on *F*^2^	Primary atom site location: structure-invariant direct methods
Least-squares matrix: full	Secondary atom site location: difference Fourier map
*R*[*F*^2^ > 2σ(*F*^2^)] = 0.059	Hydrogen site location: inferred from neighbouring sites
*wR*(*F*^2^) = 0.163	H-atom parameters constrained
*S* = 1.05	*w* = 1/[σ^2^(*F*_o_^2^) + (0.0623*P*)^2^ + 0.3001*P*] where *P* = (*F*_o_^2^ + 2*F*_c_^2^)/3
2534 reflections	(Δ/σ)_max_ = 0.012
165 parameters	Δρ_max_ = 0.18 e Å^−^^3^
0 restraints	Δρ_min_ = −0.21 e Å^−^^3^

### Special details

**Table:**

Geometry. All e.s.d.'s (except the e.s.d. in the dihedral angle between two l.s. planes) are estimated using the full covariance matrix. The cell e.s.d.'s are taken into account individually in the estimation of e.s.d.'s in distances, angles and torsion angles; correlations between e.s.d.'s in cell parameters are only used when they are defined by crystal symmetry. An approximate (isotropic) treatment of cell e.s.d.'s is used for estimating e.s.d.'s involving l.s. planes.
Refinement. Refinement of *F*^2^ against ALL reflections. The weighted *R*-factor *wR* and goodness of fit *S* are based on *F*^2^, conventional *R*-factors *R* are based on *F*, with *F* set to zero for negative *F*^2^. The threshold expression of *F*^2^ > σ(*F*^2^) is used only for calculating *R*-factors(gt) *etc*. and is not relevant to the choice of reflections for refinement. *R*-factors based on *F*^2^ are statistically about twice as large as those based on *F*, and *R*- factors based on ALL data will be even larger.

### Fractional atomic coordinates and isotropic or equivalent isotropic displacement parameters (Å^2^)

**Table:**

	*x*	*y*	*z*	*U*_iso_*/*U*_eq_	
O1	0.8723 (2)	0.22381 (12)	0.93777 (18)	0.0598 (6)	
N2	0.8981 (2)	0.20231 (12)	0.7148 (2)	0.0454 (5)	
H2A	0.8870	0.2226	0.6292	0.055*	
N1	0.7370 (2)	0.36401 (14)	0.6269 (2)	0.0552 (6)	
H1A	0.7730	0.3439	0.5594	0.066*	
C5	0.8472 (3)	0.24881 (15)	0.8093 (2)	0.0422 (6)	
C4	0.7615 (3)	0.32843 (16)	0.7622 (3)	0.0429 (6)	
O3	0.9219 (2)	−0.02724 (12)	0.8003 (3)	0.0796 (7)	
O5	1.2703 (2)	0.15643 (15)	0.5747 (3)	0.0843 (7)	
C7	0.8551 (3)	0.04766 (17)	0.7534 (3)	0.0522 (7)	
C3	0.6833 (3)	0.37952 (17)	0.8361 (3)	0.0546 (7)	
H3	0.6793	0.3712	0.9307	0.065*	
C6	0.9717 (3)	0.11846 (15)	0.7531 (3)	0.0482 (7)	
H6	1.0386	0.1232	0.8515	0.058*	
C9	1.2137 (3)	0.15003 (18)	0.6855 (4)	0.0585 (7)	
C8	1.0705 (3)	0.09670 (17)	0.6529 (3)	0.0546 (7)	
H8A	1.0969	0.0350	0.6622	0.065*	
H8B	1.0127	0.1072	0.5538	0.065*	
O2	0.7223 (2)	0.05805 (14)	0.7203 (3)	0.0953 (9)	
O4	1.2728 (3)	0.18044 (18)	0.8018 (3)	0.1036 (9)	
C2	0.6111 (3)	0.44624 (19)	0.7425 (4)	0.0681 (9)	
H2	0.5493	0.4900	0.7631	0.082*	
C1	0.6480 (4)	0.4351 (2)	0.6160 (4)	0.0718 (9)	
H1	0.6168	0.4709	0.5351	0.086*	
C11	0.8246 (4)	−0.1018 (2)	0.8043 (4)	0.0895 (11)	
H11A	0.7600	−0.0886	0.8652	0.134*	
H11B	0.8863	−0.1519	0.8418	0.134*	
H11C	0.7633	−0.1144	0.7084	0.134*	
C10	1.4150 (4)	0.2024 (3)	0.5989 (5)	0.1034 (14)	
H10A	1.4106	0.2562	0.6490	0.155*	
H10B	1.4344	0.2147	0.5078	0.155*	
H10C	1.4951	0.1663	0.6559	0.155*	

### Atomic displacement parameters (Å^2^)

**Table:**

	*U*^11^	*U*^22^	*U*^33^	*U*^12^	*U*^13^	*U*^23^
O1	0.0845 (15)	0.0672 (12)	0.0320 (10)	0.0113 (10)	0.0231 (9)	0.0068 (9)
N2	0.0597 (14)	0.0481 (12)	0.0324 (11)	0.0102 (9)	0.0190 (10)	0.0027 (9)
N1	0.0586 (15)	0.0614 (14)	0.0459 (13)	0.0154 (11)	0.0147 (11)	0.0060 (11)
C5	0.0451 (14)	0.0481 (14)	0.0354 (13)	−0.0034 (11)	0.0143 (11)	−0.0020 (11)
C4	0.0429 (14)	0.0483 (14)	0.0377 (13)	0.0000 (11)	0.0113 (11)	−0.0031 (11)
O3	0.0615 (14)	0.0542 (12)	0.1168 (19)	0.0094 (10)	0.0136 (13)	0.0197 (12)
O5	0.0604 (14)	0.1133 (18)	0.0853 (17)	0.0004 (12)	0.0300 (13)	0.0117 (14)
C7	0.0528 (17)	0.0549 (16)	0.0487 (16)	0.0091 (13)	0.0135 (13)	0.0059 (12)
C3	0.0487 (16)	0.0610 (17)	0.0569 (17)	0.0007 (12)	0.0192 (14)	−0.0126 (14)
C6	0.0551 (16)	0.0492 (15)	0.0398 (14)	0.0068 (12)	0.0122 (12)	−0.0003 (11)
C9	0.0544 (18)	0.0568 (17)	0.067 (2)	0.0100 (13)	0.0212 (16)	−0.0023 (15)
C8	0.0487 (16)	0.0591 (16)	0.0548 (17)	0.0081 (12)	0.0122 (13)	−0.0084 (13)
O2	0.0527 (14)	0.0739 (15)	0.156 (3)	0.0068 (11)	0.0223 (15)	0.0351 (14)
O4	0.0874 (19)	0.125 (2)	0.097 (2)	−0.0370 (15)	0.0233 (15)	−0.0456 (16)
C2	0.0446 (17)	0.0597 (18)	0.097 (3)	0.0085 (13)	0.0137 (17)	−0.0148 (17)
C1	0.069 (2)	0.070 (2)	0.073 (2)	0.0214 (16)	0.0127 (17)	0.0125 (17)
C11	0.092 (3)	0.0549 (19)	0.117 (3)	−0.0051 (17)	0.020 (2)	0.0205 (19)
C10	0.057 (2)	0.122 (3)	0.138 (4)	−0.004 (2)	0.038 (2)	0.029 (3)

### Geometric parameters (Å, °)

**Table:**

O1—C5	1.254 (3)	C3—H3	0.9300
N2—C5	1.333 (3)	C6—C8	1.526 (3)
N2—C6	1.447 (3)	C6—H6	0.9800
N2—H2A	0.8600	C9—O4	1.197 (4)
N1—C1	1.343 (3)	C9—C8	1.500 (4)
N1—C4	1.372 (3)	C8—H8A	0.9700
N1—H1A	0.8600	C8—H8B	0.9700
C5—C4	1.451 (3)	C2—C1	1.360 (4)
C4—C3	1.378 (3)	C2—H2	0.9300
O3—C7	1.317 (3)	C1—H1	0.9300
O3—C11	1.452 (4)	C11—H11A	0.9600
O5—C9	1.310 (3)	C11—H11B	0.9600
O5—C10	1.458 (4)	C11—H11C	0.9600
C7—O2	1.179 (3)	C10—H10A	0.9600
C7—C6	1.519 (4)	C10—H10B	0.9600
C3—C2	1.401 (4)	C10—H10C	0.9600
			
C5—N2—C6	121.4 (2)	O4—C9—C8	123.6 (3)
C5—N2—H2A	119.3	O5—C9—C8	112.7 (3)
C6—N2—H2A	119.3	C9—C8—C6	112.4 (2)
C1—N1—C4	109.5 (2)	C9—C8—H8A	109.1
C1—N1—H1A	125.3	C6—C8—H8A	109.1
C4—N1—H1A	125.3	C9—C8—H8B	109.1
O1—C5—N2	120.4 (2)	C6—C8—H8B	109.1
O1—C5—C4	120.1 (2)	H8A—C8—H8B	107.8
N2—C5—C4	119.5 (2)	C1—C2—C3	107.2 (2)
N1—C4—C3	107.0 (2)	C1—C2—H2	126.4
N1—C4—C5	124.3 (2)	C3—C2—H2	126.4
C3—C4—C5	128.7 (2)	N1—C1—C2	108.9 (3)
C7—O3—C11	117.4 (2)	N1—C1—H1	125.6
C9—O5—C10	116.6 (3)	C2—C1—H1	125.6
O2—C7—O3	123.8 (3)	O3—C11—H11A	109.5
O2—C7—C6	125.1 (2)	O3—C11—H11B	109.5
O3—C7—C6	111.0 (2)	H11A—C11—H11B	109.5
C4—C3—C2	107.5 (3)	O3—C11—H11C	109.5
C4—C3—H3	126.3	H11A—C11—H11C	109.5
C2—C3—H3	126.3	H11B—C11—H11C	109.5
N2—C6—C7	110.6 (2)	O5—C10—H10A	109.5
N2—C6—C8	110.2 (2)	O5—C10—H10B	109.5
C7—C6—C8	112.5 (2)	H10A—C10—H10B	109.5
N2—C6—H6	107.8	O5—C10—H10C	109.5
C7—C6—H6	107.8	H10A—C10—H10C	109.5
C8—C6—H6	107.8	H10B—C10—H10C	109.5
O4—C9—O5	123.7 (3)		
			
C6—N2—C5—O1	5.4 (4)	O2—C7—C6—N2	3.4 (4)
C6—N2—C5—C4	−174.2 (2)	O3—C7—C6—N2	−174.5 (2)
C1—N1—C4—C3	0.5 (3)	O2—C7—C6—C8	−120.3 (3)
C1—N1—C4—C5	177.2 (2)	O3—C7—C6—C8	61.8 (3)
O1—C5—C4—N1	175.5 (2)	C10—O5—C9—O4	1.1 (4)
N2—C5—C4—N1	−4.9 (4)	C10—O5—C9—C8	−176.2 (2)
O1—C5—C4—C3	−8.5 (4)	O4—C9—C8—C6	26.0 (4)
N2—C5—C4—C3	171.0 (2)	O5—C9—C8—C6	−156.8 (2)
C11—O3—C7—O2	3.1 (5)	N2—C6—C8—C9	73.9 (3)
C11—O3—C7—C6	−178.9 (3)	C7—C6—C8—C9	−162.1 (2)
N1—C4—C3—C2	0.2 (3)	C4—C3—C2—C1	−0.8 (3)
C5—C4—C3—C2	−176.3 (2)	C4—N1—C1—C2	−1.0 (3)
C5—N2—C6—C7	76.9 (3)	C3—C2—C1—N1	1.1 (4)
C5—N2—C6—C8	−158.1 (2)		

### Hydrogen-bond geometry (Å, °)

**Table:**

*D*—H···*A*	*D*—H	H···*A*	*D*···*A*	*D*—H···*A*
N1—H1A···O1^i^	0.86	1.96	2.804 (3)	167
N2—H2A···O1^i^	0.86	1.99	2.845 (3)	176

Symmetry codes: (i) *x*, −*y*+1/2, *z*−1/2.
